# Fibrillary Glomerulonephritis and Monoclonal Gammopathy: Potential Diagnostic Challenges

**DOI:** 10.3389/fonc.2022.880923

**Published:** 2022-05-25

**Authors:** Yi Da, Giap Hean Goh, Titus Lau, Wee Joo Chng, Cinnie Yentia Soekojo

**Affiliations:** ^1^ Division of Nephrology, Department of Medicine, National University Hospital, Singapore, Singapore; ^2^ Department of Pathology, National University Hospital, Singapore, Singapore; ^3^ Department of Haematology-Oncology, National University Cancer Institute, Singapore, Singapore

**Keywords:** fibrillary glomerulonephritis (FGN), monoclonal gammopathy of renal significance (MGRS), DnaJ homolog subfamily B member 9 (DNAJB9), monoclonal gammopathy, dysproteinemia

## Abstract

Fibrillary glomerulonephritis (FGN) is a rare glomerular disease featured by the randomly arranged 12- to 24-nm fibrils under electron microscopy (EM). Up to 10% of FGN patients have monoclonal gammopathy. However, distinguishing between FGN as monoclonal gammopathy of renal significance (MGRS) and FGN from other causes with incidental monoclonal gammopathy of undetermined significance (MGUS) can be challenging, as the current way of demonstrating monoclonality is flawed due to (1) the suboptimal sensitivity of kappa staining by immunofluorescence in frozen tissue (IF-F) as compared to pronase-digested paraffin sections (IF-P), causing incorrect labeling of light chain restriction; (2) the unavailability of immunoglobulin G (IgG) subtyping in some centers; and (3) the unavailability of tests demonstrating the monoclonality of highly variable VH or VL domains in immunoglobulin structures in clinical use. The discovery of DnaJ homolog subfamily B member 9 (DNAJB9) allows diagnosis for FGN with less reliance on EM, and the summary of recent studies revealed that genuine MGRS is extremely rare among FGN. Further research integrating IF-P, IgG subtyping, VH or VL domain monoclonality confirmation, and DNAJB9 as diagnostic modalities, with corresponding clinical data including treatment response and prognosis, is required for a better understanding of this subject.

Fibrillary glomerulonephritis (FGN) is a rare glomerular disease first described by Rosenmann and Eliakim in 1977 (1). It is identified by pathological findings of glomerular accumulations of randomly arranged, straight fibrils measuring 12–24 nm in thickness under electron microscopy (EM) (2). These fibrils accumulate in the mesangium, glomerular basement membranes, or both. Being a rare entity, comprising only 0.5%–1% of native kidney biopsies (2), FGN has a great variety in terms of its etiology, clinical manifestations, and light microscopic appearance (3). Although FGN is mostly acquired, the presence of familiar FGN has also been recognized lately (4, 5). The renal prognosis is generally poor, with nearly half of the patients progressing to end-stage renal disease within 4 years (6). After progressing to end-stage kidney disease (ESKD), patients with FGN appear to have comparable survival outcome to other ESKD causes (7). Most cases of FGN show mesangial expansion with or without GBM duplication and are less commonly associated with endocapillary hypercellularity and crescentic glomerulonephritis. On immunofluorescence, the fibrils/deposits typically show “smudgy” granular staining for immunoglobulin G (IgG), which can be polyclonal, oligoclonal, or monoclonal and complement (predominantly C3 and, in rare situations, C1q). The recent advances in FGN-related clinical research, including the discovery of DnaJ homolog subfamily B member 9 (DNAJB9), is a useful tool for a prompt diagnosis of FGN with less reliance on EM. In this article, we review recent advances in FGN and its relationship with monoclonal gammopathy.

## FGN and Monoclonal Gammopathy: What Is the Relationship?

Studies have shown that approximately 10% of FGN patients have monoclonal gammopathy. In a recent update of FGN from the United States by combining 187 FGN cases from Columbia University, Mayo Clinic, and the University of North Carolina, 13% of FGN patients had dysproteinemia, 13% had hepatitis C infection, and 11% had autoimmune disease (8). Another US cohort with 266 FGN diagnosed from the University of Washington, Oregon Health & Science University, and Stanford University yielded slightly different results of 16% association with hepatitis C, 8% with paraprotein from serum or urine, and 9% with autoimmune disease (2). In a cohort of 27 FGN patients from France, 2 (7%) had monoclonal gammopathy (3). Currently, it is standard practice to screen monoclonal gammopathy for FGN patients.

However, being associated with monoclonal gammopathy is different from monoclonal gammopathy of renal significance (MGRS). The latter requires having kidney damage caused by the produced monoclonal immunoglobulin without the underlying B cell or plasma cell clone causing systemic tumor complications or meeting any current hematological criteria for specific therapy (9). The indication for kidney biopsy in the setting of monoclonal gammopathy does not differ from the general approach to adult glomerular disease, which includes proteinuria, hematuria, and/or unexplained renal insufficiency. In a record review of 6,300 patients with monoclonal gammopathy over 5 years, only 160 (2.5%) had a kidney biopsy, with 96 patients diagnosed as MGRS (10). This indicated that MGRS is a rare entity among those with monoclonal gammopathy. On the other hand, monoclonal gammopathy of undetermined significance (MGUS) is increasingly diagnosed with an estimated crude prevalence of 3.2% in those older than 50 years from a predominantly white population (11). In the context of aging population and increasing awareness of monoclonal gammopathy, more cases of FGN or, in fact, any glomerulonephritis with incidental MGUS will be reported (12, 13). This brings up the main question in our clinical practice: how does one distinguish between FGN as MGRS, and FGN with incidental MGUS? Physicians need to be very careful as the diagnosis of MGRS carries huge implication for the treatment options and prognosis for glomerulonephritis, as clonal-related glomerulonephritis is unlikely to have spontaneous remission; it tends to respond poorly to the conventional immunosuppression without clonal-directed therapy (9), and might recur after kidney transplantation (14–16); moreover, the underlying hematological malignancy might continue to progress (17). Therefore, we need a better diagnosis approach.

## Demonstrating Monoclonality: Beyond light chain Restriction

Two possible mechanisms were proposed for the development of MGRS: direct deposition of monoclonal immunoglobulins, and indirect mechanism with activation of the alternative pathway *via* functional inhibition of complement-regulating proteins (e.g., C3 glomerulopathy and thrombotic microangiopathy). For direct mechanism, the deposition can be in glomeruli only, such as in immunotactoid glomerulonephritis and proliferative glomerulonephritis with monoclonal immunoglobulin deposits (PGNMID), whereas in light chain proximal tubulopathy (LCPT), MGRS-associated lesions involve only the proximal tubules. In cryoglobulinemic glomerulonephritis, disease involvement is mainly in the glomeruli but can occasionally affect blood vessels in the form of intravascular cryoglobulin thrombi or endovasculitis. Sometimes, all renal compartments, including glomeruli, vessels, and the tubulointerstitium, might be affected, such as in immunoglobulin-related amyloidosis and monoclonal immunoglobulin deposition disease (MIDD) (6).

In the setting of FGN, it is more of a direct mechanism, as IgG is usually present (3). Therefore, the key for diagnosing MGRS is to demonstrate monoclonality in the setting of FGN, and the monotypic pattern of FGN should match the detected monoclonal protein either in the serum or in urine. The commonly used term is light chain restriction, which refers to the presence of 1 light chain only, or the presence of staining for 1 light chain with 2+ intensity (scale of 0–3+) and at most trace staining for the other light chain on routine frozen immunofluorescence (IF-F) (18); this unfortunately does not take the heavy chains into consideration, and IgG subtyping is not universally done. A true “monotypic” immunoglobulin (Ig) should have the same light chain, heavy chain, and subtype, e.g., IgG1 kappa. However, being true monotypic does not prove monoclonal origin. In the structure of Ig, VH and VL domains are highly variable; it is possible that polyclonal Ig has the same light chain and IgG subtype but with a different VH or VL domain. Therefore, the best way to prove genuine monoclonality is by either epitope-specific antibody or amino acid sequencing of the VH and VL domains (19), which are not carried out routinely for clinical use. As a result, it is often the case that MGRS is diagnosed purely based on light chain restriction from immunofluorescence, and this is suboptimal.

Recently, there has been another debate on the different methods of demonstrating light chain restriction on immunofluorescence. Using frozen tissue for immunofluorescence is the default choice for most laboratories, and in most cases, immunofluorescence on pronase-digested paraffin sections (IF-P) is not warranted. However, this salvage method is useful especially when there is insufficient glomerulus in the frozen tissue or when “masked” deposits are suspected. Depending on the antigen tested, the intensity of staining by IF-P is in general equal to or weaker than that by IF-F; for C3, IF-P was less sensitive in all disease categories; for IgG, IF-P was less sensitive in membranous glomerulopathy or anti-glomerular basement membrane disease; however, the kappa light chain staining was more sensitive by IF-P, as compared to IF-F, in light chain proximal tubulopathy (18). This might be due to the extensive intracellular crystallization of the light chain protein rendering the antigenic sites inaccessible to antibody binding by IF-F (20). Knowing this, it is not surprising that when Said et al. re-examined FGN cases previously diagnosed by IF-F with IF-P, they found that 15 cases with light chain restriction by IF-F turned out to have no light chain restriction by IF-P, and out of the 15 cases with apparent lambda restriction by IF-F, 14 were found to have both kappa and lambda when tested by IF-P; this finding was similar to the previous study, indicating that IF-P might have better sensitivity for kappa (21). These patients had masked polyclonal deposition. The light chain monotypism by standard IF-F was false. In addition, 7 out of the 15 cases with masked polyclonal deposition also had IgG subclass restriction of IF-F (21). Therefore, adding IgG subclass staining to standard IF-F will not help this distinction, but rather, confirming the monotypism with IF-P should be prioritized. This further challenged the traditional way of diagnosing MGRS by light chain restriction, as IF-F tends to mask the presence of kappa, leading to incorrect labeling of light chain restriction.

## DNAJB9 Reshapes the Diagnosis of FGN

The breakthrough discovery of DNAJB9 may reshape our understanding of FGN. DNAJB9 was found in the glomeruli in kidney biopsy specimens using liquid chromatography and data-dependent tandem mass spectrometry (22). It is the fourth most abundant protein in FGN glomeruli based on proteomic content analysis (23). DNAJB9 is a heat-shock protein in the endoplasmic reticulum and is involved in the endoplasmic reticulum stress/unfolded response pathway, but local activation of the unfolded response pathway does not seem to drive the pathogenesis of FGN. The mechanism involved is still poorly understood; a proposed mechanism involves an increased amount of circulating DNAJB9, with an additional autoantibody response in glomerulus resulting in the abundance of DNAJB9 (24). Traditionally, EM is mandatory for FGN diagnosis but EM is not widely available (6). Nasr et al. developed DNAJB9 immunohistochemistry (IHC) and found high sensitivity and specificity for FGN (21). DNAJB9 allows a prompt diagnosis and alleviates reliance on EM (25). It also helped in making the distinction of FGN from amyloidosis, as some FGN may have Congo red positivity (26), and from other diagnostically challenging cases due to morphologically early or advanced features, or limited glomeruli for immunofluorescence or EM (2). With the discovery of DNAJB9 in FGN, we expect to see more FGN being diagnosed. There might be cases that were previously diagnosed as FGN but were in fact immunotactoid glomerulonephritis or amyloidosis, or *vice versa*, after integrating DNAJB9 into FGN diagnosis. A cohort of Ig-negative FGN was reported, which further challenges the previous FGN definition (27).

One step forward from DNAJB9 as IHC for histology testing, serum DNAJB9 level is being evaluated as a potential non-invasive biomarker for the diagnosis of FGN. This could be an advancing step towards a departure from kidney biopsy being the gold standard in diagnosing FGN lesions. Recent studies revealed that serum DNAJB9 levels accurately predicted FGN with a sensitivity of 67% and a specificity of 98%, with positive and negative predictive values of 89% and 95%, respectively (28). The main limitation of serum DNAJB9 as a non-invasive diagnostic tool for FGN includes the overlap of serum DNAJB9 range between FGN and non-FGN cases, and its inverse relationship with the estimated glomerular filtration rate (eGFR), for which the raised serum DNAJB9 level could partly be due to low eGFR (29). Given the unclear pathogenesis, the role of DNAJB9 in disease activity and treatment response is not fully understood. In the recent pilot study of using rituximab to treat FGN, there was no significant change in serum DNAJB9 levels before and after treatment (30).

## FGN is Rarely a Genuine MGRS

We believed that FGN was a type of MGRS since the early days when FGN was first recognized as a unique entity, as the apparent association with monoclonal IgG and kappa light chain deposition was highlighted (31). However, IF-P may challenge the presence of light chain restriction; IgG subtyping may challenge the monotypic nature of Ig; furthermore, monotypic Ig may not be monoclonal without confirming the VH or VL domains, and the diagnosis of FGN may be challenged by the discovery of DNAJB9. After taking all these advances into consideration, it appears that genuine MGRS is extremely rare among FGN, supported by several recent studies (**Table 1**); in fact, FGN was no longer emphasized in the latest MGRS disease category (17).

**Table 1:** Recent studies for FGN patients with status of monoclonal gammopathy, histologic diagnostic tools, and possible MGRS cases summarized.

Patient cohort	Monoclonal gammopathy detected (%)	Apparent monotypic *	Use of IF-P	Use of IgG subtyping	MGRS	DNAJB9
**266 cases with FGN** (2)	7.5%	6%	Not done	Not done	1 potential case with positive DNAJB9. Loss to follow-up.	Done in selected cases. 100% (25/25) diagnostically challenging cases and 23/29 cases with atypical features showed positive DNAJB9.
**214 cases including 84 FGN** (21)	4.8% (4 out of 84 FGN)	7%	Only when inadequate glomeruli with frozen tissue	Not done	Unable to comment.	Done for all. 97.6% (82/84) showed positive DNAJB9.
**35 apparent monotypic FGN cases** (32)	8.6% (3 out of 35 apparent monotypic FGN patients)	100%	Yes; 15/35 no longer showing light chain restriction after IF-P	Yes; 18 out of 29 were monotypic	1 case. However, complete clinical information is not available.	Done for all. 100% (35/35) showed positive DNAJB9.
**36 cases of fibrillar IgG deposits and light chain restriction** (18)	17% (5 out of 29, excluded 7 cases with no IgG subtype or no DNAJB9)	100%	Yes; IF-P unmasked kappa in 3 out of 29 cases	Yes; 13 out of 29 were monotypic	1 possible but incidental finding; main change in the biopsy was ANCA related.	Done for 29 cases. 72.4% (21/29) showed positive DNAJB9.

FGN, fibrillary glomerulonephritis; IF-P, immunofluorescence by pronase-digested paraffin sections; IgG, immunoglobulin G; MGRS, monoclonal gammopathy of renal significance; DNAJB9, DnaJ homolog subfamily B member 9; ANCA, antineutrophil cytoplasmic antibody. *Apparent monotypic referred mainly to light chain restriction with the traditional use of IF-F as the default method; IgG subtyping may or may not be performed.

The association between DNAJB9 positivity and MGRS is uncertain. From the study of Said et al. consisting of 35 cases of DNAJB9-positive FGN, the vast majority were not associated with MGRS (32). In an interesting cohort of protocol biopsy of 14 FGN post-renal transplant, 3 cases were with pre-transplant monoclonal gammopathy and did not recur with a follow-up of 4.4 years post-transplant. Notably, all 3 cases were DNAJB9-positive, with polytypic IgG, suggesting the diagnosis against MGRS, but rather FGN with incidental MGUS (33). In fact, registry data from Australia and New Zealand revealed that ESKD patients with FGN did not have a worse renal-allograft survival post-transplant as compared to patients with other causes of ESKD (7). The findings infer that it may be very rare for FGN, especially DNAJB9-positive FGN, to be genuine MGRS. Unfortunately, among the majority of cases summarized, there is a lack of data on clinical progress, treatment response to conventional immunosuppression, clonal targeted treatment, and kidney transplant. As the most difficult aspect of studying MGRS-associated diseases is their rarity, moving forward, animal models might be another way to better understand such diseases, especially with recent breakthrough advances in successful animal models in certain MGRS types (34).

In conclusion, recent advances in proving monoclonality, such as IF-P, IgG subtyping, epitope-specific antibody or amino acid sequencing for the VH or VL domain, and the discovery of DNAJB9 as a sensitive and specific marker for FGN, might potentially challenge the diagnosis of MGRS among FGN cases. Further research integrating the above-mentioned diagnostics, with corresponding clinical data, especially treatment response and prognosis, is needed for a better understanding of this subject.

## Author Contributions

Conceptualization and final editing: YD and CS. Manuscript preparation: YD, GG, TL, WC, and CS. All authors contributed to the article and approved the submitted version.

## Funding

WC is supported by National Medical Research Council (NMRC) Singapore Translational Research (STaR) Investigatorship. This research is partly supported by the National Research Foundation Singapore and the Singapore Ministry of Education under the Research Centers of Excellence initiative as well as the RNA Biology Center at the Cancer Science Institute of Singapore, NUS, as part of funding under the Singapore Ministry of Education’s Tier 3 grants, grant number MOE2014- T3-1-006. CS is supported by NMRC Fellowship.

## Conflict of Interest

The authors declare that the research was conducted in the absence of any commercial or financial relationships that could be construed as a potential conflict of interest.

## Publisher’s Note

All claims expressed in this article are solely those of the authors and do not necessarily represent those of their affiliated organizations, or those of the publisher, the editors and the reviewers. Any product that may be evaluated in this article, or claim that may be made by its manufacturer, is not guaranteed or endorsed by the publisher.
